# Hemolysin-Producing Strains among Diarrheagenic *Escherichia coli* Isolated from Children under 2 Years Old with Diarrheal Disease

**DOI:** 10.3390/pathogens9121022

**Published:** 2020-12-04

**Authors:** Anca Mare, Adrian Man, Felicia Toma, Cristina Nicoleta Ciurea, Răzvan Lucian Coșeriu, Camelia Vintilă, Adrian Cornel Maier

**Affiliations:** 1 Department of Microbiology, Pharmacy, Sciences and Technology, George Emil Palade University of Medicine, 540142 Târgu Mureș, Romania; anca.mare@umfst.ro (A.M.); adrian.man@umfst.ro (A.M.); felicia.toma@umfst.ro (F.T.); 2 Infectious Diseases Laboratory, Mureș County Clinical Hospital, 540233 Târgu Mureș, Romania; lucian-razvan.coseriu@umfst.ro (R.L.C.); camelia.vintila@spitaljudeteanmures.ro (C.V.); 3 Department of Urology, Faculty of Medicine, University “Dunarea de Jos” Galați, 800008 Galați, Romania; adrian.maier@ugal.ro

**Keywords:** diarrheagenic *Escherichia coli*, *hlyA*, MDR

## Abstract

Even if serotyping based on O antigens is still routinely used by most laboratories for the detection of diarrheagenic *Escherichia coli*, this method can provide false-positive reactions, due to the high diversity of O antigens. Molecular methods represent a valuable tool that clarifies these situations. In the Bacteriology Laboratory of Mureș County Hospital, between May 2016 and July 2019, 160 diarrheagenic *E. coli* strains were isolated from children under 2 years old with diarrheic disease. The strains were identified as Shiga toxin-producing *E. coli* (STEC)/enteropathogenic *Escherichia coli* (EPEC) via agglutination with polyvalent sera. STEC strains were serotyped using monovalent sera for serogroup O157. Simplex PCR was performed on the strains to determine the presence of the *hlyA* gene, and, for the positive ones, the hemolytic activity was tested. Antibiotic susceptibility of the identified diarrheagenic *E. coli* strains was also investigated. STEC strains were the most frequently identified (49.1%), followed by EPEC (40.2%). The *hly*A gene was identified in 12 cases, representing 18.2% of the STEC strains. Even if the extended-spectrum β-lactamase (ESBL)-producing strains represented only 10%, a relevant percentage of multidrug-resistant (MDR) strains (24%) was identified.

## 1. Introduction

Even, if in industrialized regions, medical settings and health education are available for almost everyone, diarrheal disease is still an important health issue, especially for children. Most children under 3 years suffer one or two episodes of diarrhea every year or an annual average of three episodes until 5 years. The etiology is remarkably diverse—viral (rotavirus, norovirus, astrovirus), bacterial (*Salmonella* spp., *Shigella* spp., *Campylobacter* spp., *Yersinia* spp., diarrheagenic *Escherichia coli, Clostridioides difficile*), parasitic (*Giardia lamblia*), or fungal (*Cryptosporidium* spp., *Entamoeba histolytica*). From the clinical point of view, the etiology is important, although the basic treatment is similar in all cases (rehydration, antidiarrheal medication, and probiotics). Because the antimicrobial treatment is not usually recommended for acute diarrhea in children, it is difficult to decide the etiological diagnosis and when antibiotic treatment is needed [1,2,3,4,5].

Most of the available guidelines recommend that, for acute diarrhea, diagnostic tests should be performed only in severe cases, when fever, acute abdominal pain, and stools with mucus or blood appear. The bacterial agents that are involved in most cases are *Salmonella* spp., *Shigella* spp., *Campylobacter* spp., *Yersinia* spp., *Clostridioides difficile,* and diarrheagenic *Escherichia coli*, but this etiology is influenced by the geographical area and, of course, by the socioeconomic status of the region. *Vibrio cholerae* is still a concern in developing countries, but *Shigella* spp. is the most common etiology for diarrhea in these regions. In Europe, the etiology is different, where *Campylobacter* spp., *Salmonella* spp., EPEC (enteropathogenic *Escherichia coli*), and EAEC (enteroaggregative *Escherichia coli*) are more frequently involved [6,7].

For the identification of the bacterial enteric pathogens, most laboratory protocols are clear; however, there is substantial debate regarding those for the identification of diarrheagenic *E. coli* (DEC). Classic protocols suggest that serotyping with polyvalent and monovalent antisera are the most approachable options; however, several studies proved that this technique is laborious and costly, while it can give false-positive reactions due to cross-reactivity. The most recent guidelines recommend the usage of molecular diagnostic techniques for clarifying these diagnostics. Even if molecular diagnostics methods are rapid, with a high sensibility and specificity, and even if their costs decreased in the last few years, their availability is still limited (expensive equipment, untrained personnel) [8,9,10].

Antibiotic therapy is not routinely recommended, and it is only to be applied in severe cases, immunocompromised patients, cholera, dysenteric stools (shigellosis, campylobacteriosis, nontyphoidal salmonellosis). Furthermore, in some cases, antibiotic treatment could be indicated to reduce the severity and duration of the symptoms, as well as bacterial transmission. For many reasons, even in developed countries, antibiotic treatment is still recommended more often than necessary (as a function of fever, prolonged hospitalization, persisting symptoms, or the type of physician). Even though the antibiotic resistance of diarrheagenic pathogens was described a long time ago, it is still a major concern for physicians around the world, as the resistance mechanisms are adapting and changing according to the exposure of the enteric pathogens to different antibiotics [5,11,12].

## 2. Results

In the study period, a total of 2516 fecal samples were processed for bacterial diagnostic in the Microbiology Department of the Mureș County Clinical Hospital Laboratory. The study was conducted between spring 2016 and the late summer of 2019; thus, the samples were only collected throughout the year in 2017 and 2018. In 2017, a total of 655 samples were processed for the bacterial etiology of the diarrheic syndrome. In 2018, a larger number of samples were processed (788).

Half of the processed samples (1360, 52%) were from the Pediatric Departments of the Mureș County Clinical Hospital. From the pediatric samples processed in the study period, 60.4% (822) cases were from children under 2 years old.

The gender distribution of the 822 children under 2 years old was slightly similar: 455 males (55.3%) and 367 (44.7%) females, with a mean age of 6.3 months. The most frequent request for bacterial diagnosis of the diarrheic syndrome in children under 2 years old was for 12 mnth old children (200, 25.3%), followed by requests for 1 and 8 months old children (46 each, 5.8%). On the opposite side, fewest requests were for 21 (7, 0.89%) and 23 (9, 1.1%) month old children, as well as those under 1 month old (10, 1.3%).

Mixed bacterial cultures were observed in 97.1% (798) of the 822 samples; for the remaining samples (24, 2.9%), there was no bacterial growth after 24 h of incubation.

In 21.5% of cases (177), bacterial etiology was identified for diarrheic syndrome in children under 2 years old. Two bacterial agents were identified from the same person in only two cases (*Salmonella* spp. were associated with EPEC in both cases).

DEC was the predominant etiological agent, identified in 160 cases (89.4%). Shiga toxin-producing *E. coli* (STEC) strains were the most frequently identified (88 cases, 49.2%), followed by EPEC (72 cases, 40.2%), *Salmonella* spp. (17 cases, 9.5%), and *Shigella* spp. and *Yersinia* spp., both with only one case (0.55%).

**Figure 1:**
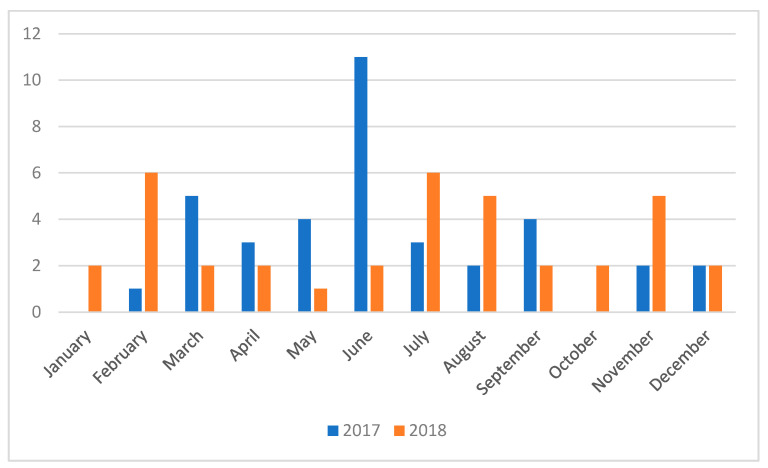
The seasonal distribution of diarrheagenic *Escherichia coli* (DEC) cases in 2017 and 2018.

There were no differences between the number of cases of DEC in 2017 and 2018 (37 cases each year). The distribution of the cases was homogeneous throughout the months of the 2 years, with three exceptions (January and October 2017—0 cases, and June 2017—11 cases) (Figure 1).

Throughout the whole study period, the DEC strains were stored by freezing (−70 °C) for further investigation. At the end of the study, only 130 strains were successfully revitalized. From these, 66 (50.8%) strains agglutinated with polyvalent pool 1 antisera (STEC), 29 (22.3%) strains agglutinated with pool 2 antisera (EPEC), and 35 (26.9%) strains agglutinated with pool 3 antisera (EPEC).

From the 66 strains with positive agglutination for pool 1 polyvalent antisera, 18 strains agglutinated with monovalent O157 antisera.

**Figure 2:**
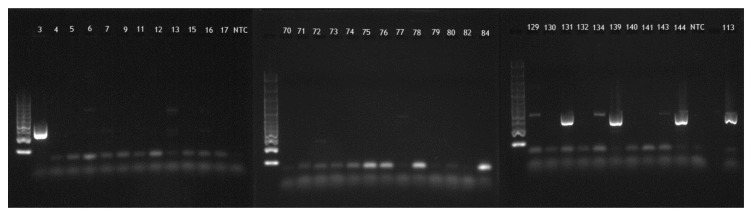
Simplex PCR electrophoresis gel representative images; strains 3, 131, 139, 144, and 113, showing positive bands for *hly*A gene, corresponding to 361 bp.

After performing simplex PCR for the 130 revitalized DEC strains (Figure 2), the *hly*A gene was identified in 12 cases, confirming that these strains were enterohemorrhagic. All 12 strains were found for STEC, representing 18.2% of the STEC strains (9.2% of the total 130 DEC strains). Only one of the strains positive for *hly*A was serotype O157.

**Figure 3:**
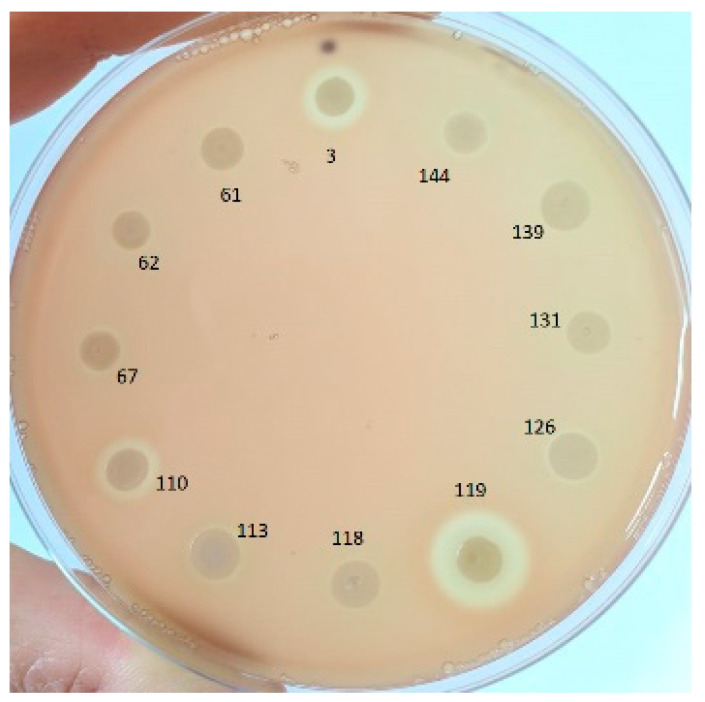
The hemolytic activity of the 12 *hly*A-positive strains.

The hemolytic activity was tested for the 12 *hly*A-positive strains (Figure 3). All strains presented hemolytic activity of different degrees (from a small and diffuse hemolysis zone surrounding the bacterial growth to a large, clear, well-defined hemolysis area).

**Figure 4:**
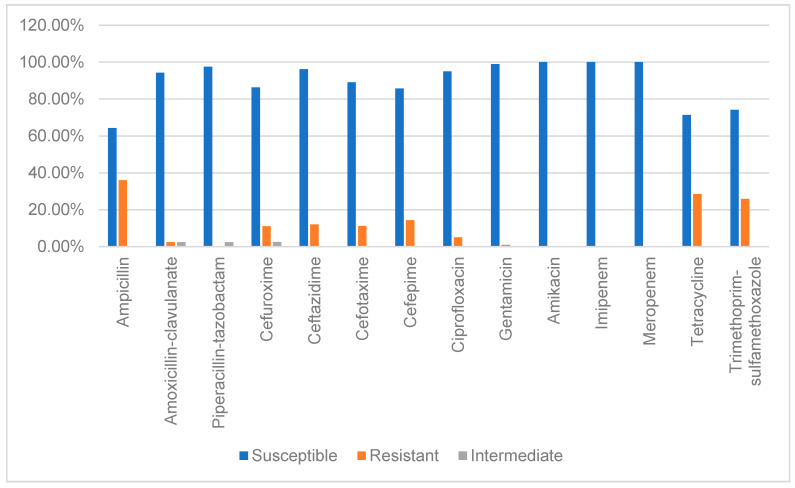
Antibiotic susceptibility of the tested DEC strains isolated from children under 2 years old.

All the tested strains fully preserved their susceptibility to carbapenems. The same situation was observed in the case of the third-generation aminoglycosides (amikacin); in terms of the first generation of aminoglycosides (gentamicin), only one strain was resistant. For the combination of beta-lactam antibiotics and beta-lactamase inhibitors, the sensitivity rates were over 94%. The sensitivity to cephalosporins was lower, between 85.7% (cefepime) and 96.2% (ceftazidime). The highest rates of resistance were reported in the case of ampicillin (36.1%), tetracycline (28.6%), and trimethoprim/sulfamethoxazole (25.9%) (Figure 4). Ten percent (13) of the tested strains were extended-spectrum beta-lactamase producers. The multidrug-resistant (MDR) strains represented 24% of the strains included in our study. All the *hly*A-positive strains were fully susceptible to all tested antibiotics, except for one strain (119), which was an MDR strain.

## 3. Discussion

Diarrheal disease is still a major concern for public health departments, no matter the age of the patients. Even if the etiology of the diarrhea is more likely to be discovered in adults [13], diarrheic agents can also be identified in children, especially young ones. Children develop a higher number of episodes and the etiology in this age group is more diverse. It was not a surprise that, in the current study, 52% of the processed samples were received from pediatric departments, with 60.4% of these samples being collected from children under 2 years old (with a mean age of 6.3 months). These were just the requests for bacterial diagnosis; thus, if the viral, parasitic, and fungal etiology would have been included in our study, the results could have been different, particularly with the knowledge that viral and parasitic etiology is more frequent in children than in adults [14,15].

Even if it is well recognized that viruses are the most frequent etiological agents for diarrheal disease in children, the bacterial etiology cannot be forgotten. *Shigella* spp., *Salmonella* spp., diarrheagenic *E. coli*, *Yersinia enterocolitica*, *Clostridioides difficile*, and *Vibrio cholerae* are well known enteric pathogens whose involvement in diarrheal etiology is influenced by multiple factors (geographic region, socioeconomic status, diagnostic protocols, etc.). In a European review published in 2015, nontyphoidal species of *Salmonella* were presented as the most common bacterial agent involved in diarrheal etiology in children under 1 year old, while, in children of 1–4 years old, *Salmonella* spp. were followed by *Campylobacter* spp. and *Yersinia* spp. [16]. A 15-year survey from the United Kingdom found *Campylobacter* spp. as the most common enteric pathogen and reported a decrease in *Salmonella* spp. frequency. A study from France also identified *Campylobacter* spp., followed by nontyphoidal *Salmonella* spp. and *Shigella* spp., while a Danish study, found *Salmonella* spp. as the most frequent bacterial agent, followed by *Campylobacter* spp., STEC, EPEC, and *Yersinia enterocolitica* [17,18,19]. A recent study from China found that diarrheagenic *E. coli* were the most frequently involved strains, followed by *Salmonella* spp. and *Shigella* spp. [20]. In Romania, the reports were also influenced by the geographic area. In a study from the southeastern region, *Salmonella* spp. were most frequently encountered, followed by *Shigella* spp. and *Campylobacter* spp. [21]. Another study from the central region of Romania identified *Campylobacter* spp. as the primary bacterial enteric pathogen, followed by *Salmonella* spp., *Klebsiella* spp., and EPEC; however, this study included children up to 14 years old [22]. Our study identified STEC as the predominant etiological agent, followed closely by EPEC, and by *Salmonella* spp., *Shigella* spp., and *Yersinia* spp. in smaller percentages.

Diarrheagenic *Escherichia coli* represent a homogeneous group of strains that, on the basis of their virulence mechanisms, colonization sites, and clinical symptoms, are classified into six major pathotypes: EPEC, EHEC (enterohemorrhagic *E. coli*)/STEC, EAEC, ETEC (enterotoxigenic *E. coli*), and EIAEC (enteroinvasive *E. coli*). Serotyping based on the different O and H antigens is considered the method of choice when *E. coli* strains need to be included in specific pathotypes. Because serotyping fails to identify certain serotypes or sometimes gives cross/false reactions, the most recent protocols recommend that diarrheagenic strains of *E. coli* should be included in the six pathotypes using molecular methods, detecting specific virulence factors (*stx1*, *stx2*, *hly*A, *escV, bfpB*, *eae*, *elt, estIa, estIb*, *invE*, *astA*, *aggR*, and *pic*). Even so, serotyping is still used in many laboratories because of the increased availability of commercial, ready-to-use antisera which can be adapted accordingly to the local pathology or because molecular diagnostics methods cannot be performed [23,24,25].

In the present study, 50.8% of the analyzed strains presented positive agglutination with polyvalent pool 1 antisera, which includes five serogroups (O26, O103, O111, O145, and O157) described by the producers as STEC [26]. While serogroup O157 is a recognized Shiga toxin-producing pathogen (including serotype O157:H7, a strain that also produces hemolysin), other serogroups such as O26 and O111 include serogroups from STEC, as well as from EPEC. The STEC strains that produce both Shiga toxin and hemolysin can cause severe syndromes, such as HUS (hemolytic uremic syndrome) [27,28]. For this reason, the production of hemolysin was further investigated. Even if the STEC strains were the only ones that presented the specific gene for *hly*A, their prevalence was high (18.2% of the STEC strains; 9.2% of the total 130 DEC strains). It is important to emphasize the fact that these strains are present in the etiology of diarrheic syndrome in children under 2 years old. It reminds us once again that the bacterial etiology of the diarrheic disease needs to be rigorously evaluated, because of the rare, but grievous possibility of it evolving as a life-threatening condition.

The therapeutic protocols for the management of diarrheal episodes in children recommend antimicrobial treatment only in severe cases. Regardless, it is a well-known fact that several practitioners still prescribe antibiotics for mild cases of diarrhea for various reasons (reducing the symptoms, pleasing the patients, underestimating the side-effects, and even reducing the consultation time) [11,12,29,30,31]. The situation is similar in Romania, where the extensive use of antibiotics and their difficult monitoring are recognized causes for the high incidence of MDR strains [32,33,34].

Antibiotic resistance among *E. coli* strains is distributed worldwide, with substantial variations across geographic regions, as an outcome of different control practices and antibiotic-prescribing practices. In 2011, the European Center for Disease Prevention and Control (ECDC) presented, in their annual surveillance report, low rates of antibiotic resistance of *E. coli* strains among northern European countries (Sweden, Norway), while, in the southern European countries, the resistance rates were higher (Italy, Cyprus, Bulgaria, Romania). The same pattern of resistance was presented in the ECDC report from 2018 [35,36]. Our study also identified a high rate of resistance against ampicillin (36%), tetracyclines (28.6%), and trimethoprim/sulfamethoxazole (25.9%), and medium resistance rates against cephalosporins (11–14%). The same ECDC reports presented low rates of carbapenem resistance (0.04% in 2011, under 1% in 2018), proving that these strains are slowly but consistently spreading among European countries. Our study did not identify carbapenem-resistant strains, but this resistance mechanism was previously reported in our geographical area [34,37]. The MDR strains represented 24% of the strains included in the current study, correlating with the data presented in the abovementioned ECDC reports for our country (over 10% in 2010, around 30% in 2018). An interesting result was that the *hly*A-positive strain that presented the highest hemolytic activity (with the largest, clearest hemolysis area around the bacterial growth) was an MDR strain.

The differences between the reported percentages of *E. coli* extended-spectrum β-lactamase (ESBL)-producing strains (ranging from 4.7% in a study from northern European countries to 7.69% in a Spanish study, 15.3% in a European study, and over 50% in some Asiatic and African regions) can be explained by factors such as differences in the laboratory methodology or different antibiotic prescription patterns [38,39,40,41]. Our study identified 10% extended-spectrum beta-lactamase-producing strains, smaller than the proportion presented in other local studies [42,43,44].

In addition to the selection of resistance mechanisms, disruption of the commensal intestinal flora is another documented side-effect of excessive and prolonged antibiotic treatment (prescribed with or without real motivation). From the fecal samples processed from children under 2 years old, the current study identified 2.9% samples with no bacterial growth, probably because of previous or ongoing antibiotic treatments. The percentage of “sterile” samples was low because the samples were collected from hospitalized children, treated by pediatricians, which are more circumspect and prudent with unnecessary antibiotic prescriptions than the general practitioners.

## 4. Materials and Methods

A retrospective study was conducted, including 822 samples of diarrheic stools from children under 2 years old, received in the Microbiology Department of the Mureș County Clinical Hospital Laboratory, Romania, between April 2016 and August 2019 (with hospital approval). The fecal samples were processed with routinely standard protocols used in the laboratory for stool cultures (culturing on selective media and phenotypical identification) to identify the bacterial enteric pathogens *Shigella* spp., *Salmonella* spp., EPEC, EHEC, *Yersinia enterocolitica*, and *Vibrio cholerae*. A minimum number of five lactose-positive colonies (randomly chosen) were isolated on nonselective media and further identified for the suspicion of EPEC by agglutination using polyvalent sera (SSI Diagnostica, Denmark, OK O pool 1—STEC, 2—EPEC, 3—EPEC antisera). The strains that were positive for agglutination with pool 1 antisera were further tested with monovalent O157 antisera. Lastly, the antibiotic susceptibility of the identified enteric pathogen strains was investigated, using qualitative (Kirby–Bauer/disc diffusion method) or quantitative techniques (minimum inhibitory concentration, Vitek 2 Compact 30), following the CLSI (Clinical Laboratory Standards Institute) recommendations [45]. An isolate was considered MDR when it presented resistance toward more than three antibiotics from different classes.

### 4.1. PCR

Simplex PCR was performed for the 130 DEC strains that were revitalized at the end of the study to determine the presence of the *hly*A gene, specific for enterohemorrhagic strains.

The boiling method was performed for the extraction of the genomic DNA. One bacterial colony was vortexed, in a 2 mL tube, with DNase-free water (500 µL). After the mixture was boiled for 20 min, it was immediately placed in a freezer (−20 °C, 10 min). Afterward, the suspension was centrifugated for 5 min (16,000× *g*, 4 °C). From the supernatant (containing the extracted DNA), 300 µL of the sample was transferred to a new sterile 2 mL tube.

For the simplex PCR, the following primers were used: *hly*A forward (F)—GTAGGGAAGCGAACAGAG and *hly*A reverse ^®^—AAGCTCCGTGTGCCTGAA. A final 25 µL volume mixture was prepared for each sample (1 µL of the genomic bacterial DNA, 1 µL of primer *hly*A F, 1 µL of primer *hly*A R, 9.5 µL of DNase free water, and 12.5 µL of DreamTaq Green^®^ PCR Master Mix 2×, ThermoFisher Scientific, CA, USA); the PCR reaction was performed under the following conditions: 4 min initial denaturation at 94 °C, 30 denaturation cycles of 45 s at 94 °C, annealing for 60 s at 55 °C, extension for 1 min at 72 °C, and a final extension for 7 min at 72 °C. The PCR products were loaded on a 1.5% agarose gel (stained with GelRed^®^). The electrophoresis was performed in 1× Tris/borate/ethylenediaminetetraacetic acid (TBE) buffer, at 100 V, for 1 h (a molecular ladder and no-template control were also used). The image was visualized and captured with the MiniBIS Pro (Bio-Imaging Systems, Jerusalem, Israel) gel documentation system.

### 4.2. Hemolytic Activity

The hemolytic activity was detected following a previously published protocol, on sheep blood agar (5% defibrinated erythrocytes, washed in PBS—phosphate-buffered saline, pH 7.4), supplemented with 10 mM calcium chloride [46]. The bacterial strains were cultured overnight in tryptic soy broth. Two microliters of the homogenized liquid culture were spotted on the blood agar plates. After 24 h of incubation at 37 °C, the presence of hemolysis surrounding the bacterial culture was visually examined.

## 5. Conclusions

In the current study, STEC was the predominant etiologic bacterial agent of diarrheal disease in children under 2 years old, followed closely by EPEC. These strains presented a high rate of resistance against ampicillin, tetracyclines, and trimethoprim/sulfamethoxazole, and medium resistance rates against cephalosporins. Even if the ESBL-producing strains represented only 10% of the total sample, a significant number of MDR strains were identified (24%). Interestingly, the *hly*A-positive strain with the highest hemolytic activity was an MDR strain.

The fact that 18.2% of the STEC strains were hemolysin producers draws attention to the importance of constantly updating the laboratory protocols for a correct and up-to-date diagnostic procedure.
